# Intravaginal practices and lactobacilli colonization among women in Accra, Ghana

**DOI:** 10.1186/s12905-015-0205-2

**Published:** 2015-06-04

**Authors:** Francisca Nana-Aba McCarthy, Nicholas Israel Nii-Trebi, Billal Obeng Musah, Richard Harry Asmah

**Affiliations:** Department of Medical Laboratory Sciences, School of Biomedical and Allied Health Sciences, College of Health Sciences, University of Ghana, Accra, Ghana

**Keywords:** Intravaginal practices, Lactobacillus colonization, Women, Accra

## Abstract

**Background:**

Intravaginal practices may affect the colonization of vaginal flora and lead to vaginal infections due to the potential effects on the vaginal environment. This study investigated the vaginal practices and their possible effects on vaginal lactobacilli flora colonization in women in Accra.

**Methods:**

A cross-sectional, descriptive single-site study was carried out on 141 women assessing medical care at the Obstetrics and Gynaecology Department of the Korle-Bu Teaching Hospital (KBTH) in Accra. Study-relevant information on participants was obtained by means of questionnaire. Vaginal swab samples were collected and processed for laboratory analyses.

**Results:**

All the participants (141/141, 100.0 %) indicated they performed intravaginal practices using various methods. Almost half (46.1 %) of these women were between the ages of 25–34 years and 65.0 % were married. Internal douching (82.3 %; p > 0.05) was the commonest practice reported. Other practices such as insertion and wiping with hands and objects, as well as use of locally prepared concoctions and certain commercial products were also reported. The reason most commonly given was for hygienic purpose (83.0 %); a few (10.6 %) did it for sexual satisfaction, while others indicated vaginal tightness (5.7 %) and wound healing (0.7 %) as reasons for their practice. No *Lactobacillus sp*. was detected in as many as 78.7 % of the sample. Association tests by the Pearson correlation analysis showed strong significant negative correlation (r = −0.954, p < 0.05) between use of traditional herbs/concoction and vaginal lactobacilli colonization; and douching being the least negatively (r = −0.601, p > 0.05) correlated practice.

**Conclusions:**

Vaginal practices were common among the women studied. A more elaborate prospective, case–control study into intravaginal practices and their impact on the health of women in Ghana should be explored.

**Electronic supplementary material:**

The online version of this article (doi:10.1186/s12905-015-0205-2) contains supplementary material, which is available to authorized users.

## Background

The lower female genital tract normally harbours mutually coexisting microflora that largely consist of lactobacilli [1, 2]. Studies have shown four distinct lactobacilli species, namely *L. crispatus, L. jensenii*, *L. gasseri* and *L. iners* to be commonly found in the lower genital tract of many women [3–5]. Of these, *L. iners* has been suggested to predominate by some studies; and it has also been described as marker of changes in the vaginal flora [6, 7]. Predominant vaginal colonization by lactobacilli is believed to be intrinsically beneficial in conferring antimicrobial protection to the vaginal epithelium against colonization by other microorganisms [8–10]. The colonization by lactobacillus microflora has also been associated with lower rates of infection with a number of sexually transmitted infectious diseases; whilst low level of vaginal lactobacilli predisposes to infections such as trichomoniasis, gonorrhoea, chlamydiosis and HIV [11–15]. However, vaginal lactobacilli dominance and the inherent antimicrobial potential fail in a number of women and their association with health and/or transition from health to disease or vice versa appear to be species-dependent [16–18]. For example, the presence of hydrogen peroxide (H_2_O_2)_-producing lactobacilli, *L. crispatus* and *L. jensenii,* has been associated with a lower occurrence of bacterial vaginosis (BV); and a longitudinal analysis of the vaginal microflora in pregnancy suggests that *L. crispatus* promotes the stability of the normal vaginal microflora while *L. gasseri* and/or *L. iners* to some extent predispose to the occurrence of abnormal vaginal microflora [19–22].

The extent of inter-individual differences in vaginal lactobacilli flora composition, and the factors associated with loss of vaginal lactobacilli remain to be fully explored. It is conceivable that non-microbial factors including menstruation, sexual intercourse as well as vaginal practices engaged in by women to manage their vaginal health and sexual life could possibly disturb the vaginal ecological conditions such as the low pH, which enable lactobacilli to inhibit overgrowth by other microorganisms in the vagina [23–25].

Intravaginal practices are commonly performed by women worldwide and varies in methods and substances used in different populations across sub-Saharan Africa [26, 27]. Methods such as internal cleansing or washing inside the vagina, which includes wiping the internal genitalia with fingers or other substances; douching, which is the pressurised shooting or pumping of water or solution into the vagina; and insertion of locally made or commercial cleansing products and objects into the vagina have been described [28]. Various studies have also recorded a variety of substances used, which include lemon juice, traditional herbs, commercially available products and detergents. Solid items applied include cloth, cotton wool, paper and stones [29, 30]. Even though these practices are undertaken for a range of purposes including hygiene, sexual satisfaction, prevention of disease or pregnancy, or merely following cultural norms [31, 32], several problems could be associated. Importantly, insertion or pushing of substances such as powders, creams, herbs, tablets, sticks, stones, pulverized rock, leaves or some local and commercial products can cause physical abrasions and potentially disrupt vaginal flora, leading to bacterial vaginosis (BV) [7, 9] and other sexually transmitted infections including HIV [26, 29, 33]. Knowledge on the occurrence of these practices and their effects on women’s health remain to be fully explored.

This study was therefore conducted to primarily describe intravaginal practices among Ghanaian women in Accra and assess possible association between observed practices and lactobacilli colonization as vaginal flora, in a single-site cross-sectional study.

## Methods

### Study area and design

The study was carried out at the Department of Obstetrics and Gynaecology and Central Laboratory of the Korle-Bu Teaching Hospital, Accra. The Korle-Bu Teaching Hospital is the largest and a major referral hospital located in the nation's capital, Accra, which offers medical services to people from any part of the country. The Central Laboratory is the hospital's biggest laboratory where all samples are sent for laboratory investigations. The study was cross-sectional. It investigated the occurrence of vaginal practices and levels of vaginal *Lactobacilli sp*. colonization in women.

### Subject selection and specimen collection

Women who met the study inclusion criteria were sampled for the investigation. All women assessing care at the Obstetrics and Gynaecology department between March and July, 2013 were approached. Participation was voluntary and reasons were not obtained from the few women who declined participation. Qualified and consenting women were recruited for the study. They were briefed about the study and a voluntary written informed consent was sought from those willing to participate. The consent, available in English, was translated into a local language (either Twi or Ga) for better understanding according to the respondent's wish. Details about the study regarding importance and potential risks were explained to all respondents. Once all questions had been answered, those willing to participate indicated consent by appending their signature or thumb print on the form. Consenting respondents who were between 18 to 50 years and were not on antibiotic medication were eligible for the study. Other exclusion criteria were pregnancy, vaginal antimicrobial usage in the previous 14 days, severe physical handicaps, menstruation and inability to speak English, Twi or Ga.

One hundred and forty one (141) women were enrolled for the study. Questionnaires were administered to obtain study-relevant information from participants. The information required included the socio-demographic characteristics (age, education, marital status); intravaginal practices (knowledge, methods, reasons, time of the practices); sexual behaviour (numbers of sex partners, frequency of sex, sex during menstruation, general condom use, use of lubricants during sex) and contraceptive use (hormonal contraception by way of oral pills, insertion of capsules or injection; insertion of intrauterine device, other method of birth control) by the participants (see Additional file 1 for full details of the study questionnaire). Data collection by the questionnaire lasted 30 minutes on average per participant.

With the assistance of a qualified nurse high vaginal swab (HVS) was taken (by using a sterile disposable speculum) at the emergency room of the Obstetrics/Gynaecology Department. The samples were placed in transport medium and transported to the Central laboratory of the Korle-Bu Teaching Hospital (KBTH) for laboratory investigation.

### Laboratory analysis

#### Microscopic examination

Initially, microscopic procedures were followed to investigate *Lactobacilli sp*. presence using wet mount preparation with normal saline. Briefly, about 1 ml of normal saline (0.85gNaCl) was dispensed into the transport medium within which the swab stick is placed and was shaken gently to allow homogenisation. A drop of the saline was placed on a labelled grease-free slide to make a wet preparation and cover-slipped. The wet preparation was examined systematically under the light microscope using 10× objective lens for scanning materials and 40× for identification of the organisms.

#### Culture and biochemical assays

Standard culture and biochemical techniques for the identification and confirmation of *Lactobacilli sp.* were followed [34]. Briefly, the swab on the stick was used to make a pool under aseptic conditions on both Blood agar and Chocolate agar plates. The inoculum was evenly streaked by use of a sterilized wire loop to ensure discrete colonies. The culture plates were incubated overnight at 37 °C. Identification of the organism was done by colonial morphology and confirmed by Gram staining and biochemical test. Colonial morphology was done by examining and grouping the colonies based on the shape, colour, border, texture, and general appearance of the colonies of bacteria on each plate. Gram staining was performed on the bacterial colonies and examined with 100× objective lens and bacteria were identified as either Gram positive or Gram negative based on the staining properties. The Gram positive cocci bacteria were identified by Catalase and Coagulase tests to confirm the presence of *Lactobacilli sp*.

### Data analysis

Results obtained from the study were entered into a database and analysed statistically using Microsoft Excel® software and Statistical Package for Social Sciences (SPSS) version 20 statistical software for windows and summaries were presented using descriptive statistics. A Pearson correlation run was used to determine any significant correlation at p < 0.05 between the intravaginal practices and the occurrence of vaginal *Lactobacilli sp.*

### Ethics

Ethical approval was obtained from the Research and Ethical Protocol Review Committee of the School of Biomedical and Allied Health Sciences, College of Health Sciences of the University of Ghana. Voluntary written informed consent was obtained from the study participants.

## Results

A total of 141 eligible females attending clinic at the Obstetrics and Gynaecology Department of the KBTH and aged between 18 and 50 years participated in the study. Findings showed that women of different age groups, marital status and educational level engaged in intravaginal practices. Almost half (46.1 %) of the participants were aged between 25–34 years. Of the number studied, at least 65.0 % were married and quite an appreciable percentage (90.8 %) had at least primary level of education (Table 1).

**Table 1:** Summary of socio-demographic characteristics of study participants

Variable	Frequency reporting with intravaginal practices	Percentage (%) occurrence
**Age (years)**		
18-24	42	29.8
25-34	65	46.1
35-44	24	17.0
45-50	10	7.1
**Marital status**		
Single	43	30.5
Married	92	65.2
Divorced	6	4.3
**Educational level**		
No formal education	13	9.2
Primary/Secondary	87	61.7
Tertiary	41	29.1
Total participant	141	100.0

The ages of the participants of the study ranged between 18 and 50 years. Close to 50 % of the 141 them were aged between 25 and 34 years. At least 65 % of the participants were married and a good number of them (128/141) had at least primary level of education

Various types of intravaginal practices recorded among participants and the reasons given by the women for the practices are shown in Table 2. Majority (82.3 %) indicated they either used water only or sometimes water and soap (douching). The rest of the participants used other methods such as commercially available creams and products, wiping with hands and objects, and the use of herbs and concoctions among others. A high percentage (83.0 %) of the participants indicated hygienic purposes as reasons for their practice. Others said they do it for sexual satisfaction (10.6 %), vaginal tightness (5.7 %) and a small number (0.7 %) indicated vaginal wound healing as the reason for engagement in the practice. Lactobacilli colonization was detected in 21.3 % of the participants.

**Table 2:** Intravaginal practices and reasons for engagement by participants

Intravaginal practices	Frequency of participants	Percentage, % (P-Value)
**Practicing**	141	100.0
Yes	0	0.0
No		
**Method**		
Water only/water and Soap	116	82.3(0.075)
Commercial product	14	9.9(0.030)
Wiping with hand/objects	4	2.8(0.044)
Herbs/concoctions	5	3.6(0.001)
Others	2	1.4(0.060)
**Reasons**		
Hygienic purposes	117	83.0
Sexual satisfaction	15	10.6
Vaginal tightness	8	5.7
Wound healing	1	0.7
Total	141	100.0

All the women studied engaged in intravaginal practices. In the Table are listed the methods they use and the reasons for their practices. Predominantly most of the participants use either water only or water with soap for the purpose of maintaining vaginal hygiene

To determine if there exists any correlation and the extent of the relationship between the various intravaginal practices and lactobacilli presence, a Pearson correlation analysis performed showed a strong significant negative correlation (r = −0.954, p < 0.05) between use of Herbs/concoction and vaginal lactobacilli colonization; followed by use of commercial products, also with a strong negative relationship (r = − 0.700, p < 0.05) with Lactobacilli occurrence. The analysis showed douching as being the least negatively correlated practice (r = −0.601, p > 0.05).

## Discussion

This study recorded some intravaginal practices among the women studied in Accra. The commonest method practiced is vaginal washing with water only or water with soap. Besides the commonest form of the practice observed, the use of other methods such as insertion and wiping with hands and objects, use of locally prepared concoctions and certain commercial products among others were also found to be practiced. The results of the association tests by the Pearson correlation analysis indicate statistically significant adverse relationship between some of the practices, especially locally prepared and some commercial products, and vaginal flora status. This study did not undertake to confirm vaginal infections and establish causality between infections and intravaginal practice. A case–control study over a period of time is needed in future to describe the effects of various intravaginal practices on vaginal health in Ghanaian women.

Considering the age groups of persons who indicated they indulge in intravaginal practices, the types of practice and the reasons given for each practice, the findings of this study are comparable to what have been described from previous studies in other populations. Of importance is the fact that most of the reports on vaginal practices are from areas of high HIV prevalence [30, 31, 35–37] and where heterosexual means accounts for the highest mode of HIV transmission [38–40]. This underscores the need for further studies into intravaginal practices in Ghana to determine association with sexually transmitted infections and inform health education.

The results of this study were interpreted and presented in the context of several limitations: data were not obtained on routine physical and pelvic examinations of women; STIs were not diagnosed and confirmed by laboratory analysis; and species level identification of lactobacilli culture isolates was not performed and so we did not attempt to correlate culture results with intravaginal practices. In addition, all the women studied indicated they engaged in intravaginal practices and thus a comparison could not be made about lactobacilli colonization between women reporting and those not reporting intravaginal practices. The study mainly presents data on occurrence of vaginal practices among women in the study site population and documents the level of colonization of lactobacilli as normal vaginal flora of women who engage in intra-vaginal practices.

## Conclusions

Various forms of intravaginal practices were engaged in by the women studied; and colonization by lactobacilli, a normal vaginal flora, among the studied women was found to be generally low. The practices were engaged in for the purposes of achieving vaginal hygiene and sexual comfort among others. In Ghana, the vaginal health implications of intravaginal practices have not been well studied and documented. This study therefore concludes that there is evidence of intravaginal practices being performed by some women in Accra and hence there is the need for further, more elaborate study to document the prevalence of intravaginal practices and clarify associations between various intravaginal practices and vaginal health in Ghanaian women.

## Additional file

Additional file 1:
**Study Questionnaire.**
