# Supplementary material for: Intravaginal practices and lactobacilli colonization among women in Accra, Ghana
Source: BMC Womens Health. 2015 Jun 4;15:46. doi: 10.1186/s12905-015-0205-2 (PMC4455987; doi:10.1186/s12905-015-0205-2)
Supplement: Additional file 1: — Study Questionnaire. [file 12905_2015_205_MOESM1_ESM.pdf]

STUDY QUESTIONNAIRE

Intravaginal practices and lactobacilli colonization among women in Accra, Ghana

ID No:……………...

Date: ……………….

Introduction

This study, being conducted here in the Korle Bu Teaching Hospital of Accra, Ghana aims to describe intravaginal practices among women and document the level of colonization of lactobacilli as normal vaginal flora of women who engage in intra-vaginal practices. The outcome is hoped to provide basis for further, more elaborate study to document the prevalence of intravaginal practices and clarify associations between various intravaginal practices and vaginal health in Ghanaian women. This questionnaire therefore mainly seeks to obtain information on demographic data, knowledge and occurrence of intravaginal practices, sexual behaviour and related lifestyle of participants. You are invited to take part in the study by answering the following questions as accurately and honestly as possible. You are assured that any information given is solely for research purpose and will be kept confidential. Thank you for your participation.

In the following questions please tick (√) as appropriate in the box provided.

SECTION A: SOCIO-DEMOGRAPHIC CHARACTERISTICS

1. To which age group do you belong?

1) <25yrs [ ]
2) 25-30 yrs.[ ]
3) 30 – 34 yrs.[ ]
4) 35 – 39 yrs. [ ]
5) 40 – 44 yrs. [ ]
6) >44yrs [ ]

2. Marital Status

1) Married [ ]
2) Separated [ ]
3) Divorced [ ]
4) Widowed [ ]
5) Cohabiting [ ]
6) Single, never married [ ]

3. What is your highest level of education?

1) Did not school/ did not complete primary [ ]
2) Primary [ ]
3) Secondary [ ]
4) Tertiary [ ]
5) Other [ ] (Please specify) ………………………………

4. Occupation

1) Unemployed [ ]
2) Professional [ ]
3) Artisan [ ]
4) Trader [ ]
5) Other [ ] (Please specify) ....

SECTION B: INTRAVAGINAL PRACTICES

5. Which type of intravaginal practices do you know about?

1) Douching [ ]
2) Cleansing with soaps [ ]
3) Cleansing with lemon juice [ ]
4) Cleansing with salt solution [ ]
5) Use of commercially available products [ ]
6) Use of traditional herbs and herbal concoction [ ]
7) Insertion of creams [ ]
8) Wiping with objects [ ]
9) Use of detergents [ ]
10) Other [ ] (Please specify)..

6. Do you engage in vaginal practices in any way for any reason?

1) Yes [ ]
2) No [ ]

If No proceed to Section C

7. Which of the practices do you engage in? Specify/describe where appropriate.

1) Douching [ ]
2) Cleansing with soaps [ ]
3) Cleansing with lemon juice [ ]
4) Cleansing with salt solution [ ]
5) Use of commercially available substances [ ]
6) Use of traditional herbs and herbal concoction [ ]
7) Insertion of creams [ ]
8) Wiping with objects [ ]
9) Use of detergents [ ]
10) Other [ ] (Please specify)...

8. How do you apply these substances?

1) Hands [ ]
2) Sticks [ ]
3) Cotton wool [ ]
4) Paper [ ]
5) Other [ ] (please specify) ....

9. What are some of your reasons for engaging in the practice?

1) Hygienic purposes [ ]
2) Sexual satisfaction [ ]
3) Vaginal tightness [ ]
4) Prevent vaginal infections [ ]
5) Wound healing [ ]
6) Other [ ] (Please specify)..

10. When do you perform intravaginal practices?

1) After having sex [ ]
2) After menstruation [ ]
3) After bathing [ ]
4) Other [ ] (Please specify)..

11. Have you ever experienced any unusual effect after performing intravaginal practice?

1) Yes [ ]
2) No[ ]

If No proceed to Section C

12. If you answered 'Yes' in (12) above, please specify the experience.

1) Itching / irritation [ ]
2) Pain [ ]
3) Vaginal infections [ ]
4) Discomfort [ ]
5) Bleeding [ ]
6) Other [ ] (Please specify) ....

SECTION C: SEXUAL BEHAVIOUR

13. How many sexual partners do you have?

1) No sex partner [ ]
2) One [ ]
3) Two [ ]
4) Three [ ]
5) More than three [ ]

14. Have you had/ changed sexual partner before?

1) Yes [ ]
2) No [ ]
3) Not applicable [ ]

If No continue from question 17.

If does not apply continue from question 21.

15. How many sex partners have you had in the past 4 weeks?

1) One partner [ ]
2) Two partners [ ]
3) Three or more [ ]

16. Averagely, how many times do you have sex?

1) Once a week [ ]
2) Once a month [ ]
3) Once every two months [ ]
4) Other [ ] (Please specify)……………………………………………………

In questions 11 – 19 please tick Yes or No.

17.Do you use condom during sex?

1) Yes [ ]
2) No [ ]

18. Do you use lubricants during sex?

1) Yes [ ]
2) No [ ]

19. Do you have sex during menstruation?

1) Yes [ ]
2) No [ ]

SECTION D: METHODS OF BIRTH CONTROL (CONTRACEPTIVE USE)

20. Which contraceptive do you use ?

1) Contraceptive pills [ ]
2) Depot Medroxyprogesterone Acetate (DMPA) [ ]
3) Intrauterine device [ ]
4) Norplant [ ]
5) None [ ]
6) Other [ ] (Please specify)……………………………………………………

SECTION E: INFECTIONS OF THE GENITAL TRACT

21. Have you had any genital tract infection before?

1) Yes [ ]
2) No [ ]

22. Which type of vaginal infection have you had before?

1) *Neisseria gonorrhoea* [ ]
2) *Chlamydia trachomatis* [ ]
3) Cervicitis [ ]
4) Bacterial vaginosis [ ]
5) *Trichomonas vaginalis* [ ]
6) Vaginal candidiasis [ ]
7) Syphilis [ ]
8) Other [ ] (Please specify)..
